# Allergen immunotherapy for allergic asthma: protocol for a systematic review

**DOI:** 10.1186/s13601-016-0094-y

**Published:** 2016-02-09

**Authors:** Sangeeta Dhami, Ulugbek Nurmatov, Ioana Agache, Susanne Lau, Antonella Muraro, Marek Jutel, Graham Roberts, Cezmi Akdis, Matteo Bonini, Moises Calderon, Thomas Casale, Ozlem Cavkaytar, Linda Cox, Pascal Demoly, Breda Flood, Eckard Hamelmann, Kenji Izuhara, Ömer Kalayci, Jörg Kleine-Tebbe, Antonio Nieto, Nikolaos Papadopoulos, Oliver Pfaar, Lanny Rosenwasser, Dermot Ryan, Carsten Schmidt-Weber, Stan Szefler, Ulrich Wahn, Roy-Gerth van Wijk, Jamie Wilkinson, Aziz Sheikh

**Affiliations:** 1 Evidence-Based Health Care Ltd, Edinburgh, UK; 2 Systematic Review at Decision Resources Group Abacus International, Oxford, UK; 3 Department of Allergy and Clinical Immunology, Faculty of Medicine, Transylvania University Brasov, Brasov, Romania; 4 Department of Pediatric Pneumology and Immunology, Charité Medical University, Berlin, Germany; 5 Food Allergy Referral Centre Veneto Region, University Hospital of Padua, Padua, Italy; 6 Wroclaw Medical University, Wrocław, Poland; 7 The David Hide Asthma and Allergy Research Centre, St Mary’s Hospital, Newport Isle of Wight, NIHR Respiratory Biomedical Research Unit, University Hospital Southampton NHS Foundation Trust, Southampton, UK; 8 Faculty of Medicine, University of Southampton, Southampton, UK; 9 Swiss Institute for Allergy and Asthma Research, Davos, Switzerland; 10 Sapienza University Rome, Rome, Italy; 11 National Heart and Lung Institute, Imperial College, London, London, UK; 12 University of South Florida, Tampa, FL USA; 13 Department of Allegy and Clinical Immunology, Sami Ulus Maternity and Children Training and Research Hospital, Ankara, Turkey; 14 Nova Southeastern University, Fort Lauderdale, FL USA; 15 University and Hospital of Montpellier and Inserm Paris Sorbonnes, Montpellier, France; 16 European Federation of Allergy and Airways Diseases Patients Association, Brussels, Belgium; 17 Children’s Center Bethel, EvKB, Bieledelf, Germany; 18 Allergy Center, Ruhr-University, Bochum, Germany; 19 Saga Medical School, Saga, Japan; 20 Hacettepe University, Ankara, Turkey; 21 Allergy and Asthma Center Westend (AAZW), Berlin, Germany; 22 Children’s Hospital La Fe, Valencia, Spain; 23 Allergy Department, 2nd Pediatric Clinic, University of Athens, Athens, Greece; 24 Department of Otorhinolaryngology, Head and Neck Surgery, University Hospital, Mannheim, Mannheim, Germany; 25 Center for Rhinology and Allergology, Wiesbaden, Germany; 26 Children’s Mercy Hospital, Kentucky, MO USA; 27 University of Edinburgh, Edinburgh, UK; 28 Technische Univ and Helmholtz Center Munich, Munich, Germany; 29 Children’s Hospital Colorado, University of Colorado School of Medicine, Aurora, CO USA; 30 Department of Pediatric Pulmonology, Charite, Berlin, Germany; 31 Section of Allergology, Department of Internal Medicine, Erasmus MC, Rotterdam, The Netherlands; 32 Pharmaceutical Group of the European Union, Brussels, Belgium; 33 Allergy and Respiratory Research Group, The University of Edinburgh, Edinburgh, UK

**Keywords:** Allergy, Allergic asthma, Allergen immunotherapy, Disease-modifying, Respiratory allergy

## Abstract

**Background:**

The European Academy of Allergy and Clinical Immunology (EAACI) is in the process of developing the EAACI Guidelines for Allergen Immunotherapy (AIT) for Allergic Asthma. We seek to critically assess the effectiveness, cost-effectiveness and safety of AIT in the management of allergic asthma.

**Methods:**

We will undertake a systematic review, which will involve searching international biomedical databases for published, in progress and unpublished evidence. Studies will be independently screened against pre-defined eligibility criteria and critically appraised using established instruments. Data will be descriptively and, if possible and appropriate, quantitatively synthesised.

**Discussion:**

The findings from this review will be used to inform the development of recommendations for EAACI’s Guidelines on AIT.

**Electronic supplementary material:**

The online version of this article (doi:10.1186/s13601-016-0094-y) contains supplementary material, which is available to authorized users.

## Background

Asthma is a major public health problem affecting over 300 million people worldwide [1]. Its prevalence and impact are particularly on the rise in urbanized regions. With a projected surge in the world’s urban population it is estimated that by 2025 an additional 100 million people may develop asthma [2]. Asthma is therefore set to become one of the world’s most prevalent chronic diseases.

Pathophysiologically asthma is a chronic inflammatory disorder of the airways leading to airflow limitation and remodeling [3]. The resulting signs and symptoms are dyspnea, cough, chest discomfort, wheezing and anxiety. Based on clinical and laboratory findings, different phenotypes have been described [4]. The pathogenesis of asthma is highly complex and several disease endotypes have been suggested [5]. This review will focus on allergic asthma. Currently there is no cure for asthma available but symptomatic control can be achieved with inhaled steroids with minimal if any side-effects. Long-acting beta-2 agonists, antileukotrienes, theophylline, anti-IgE antibodies and anticholinergic drugs can be added to achieve asthma control in more severe cases [6].

Allergen immunotherapy (AIT) is the only effective anetiological treatment for respiratory allergy, which has the potential to change the course of the disease. Its immunological mechanisms of action have been demonstrated as induction of allergen-specific immune tolerance. AIT for allergic asthma is a potential therapeutic option for well-selected patients [7].

The European Academy of Allergy and Clinical Immunology (EAACI) is in the process of developing the EAACI Guidelines for allergen immunotherapy (AIT), and this systematic review is one of five inter-linked evidence syntheses that are being undertaken in order to provide a state-of-the-art synopsis of the current evidence base in relation to evaluating AIT for the treatment of allergic asthma, allergic rhino conjunctivitis, food allergy and venom allergy, and allergy prevention, which will be used to inform the formulation of key clinical recommendations. The focus of this review is on assessing the effectiveness, safety and cost-effectiveness of AIT in the management of allergic asthma.

## Methods

### Search strategy

**Fig. 1:**
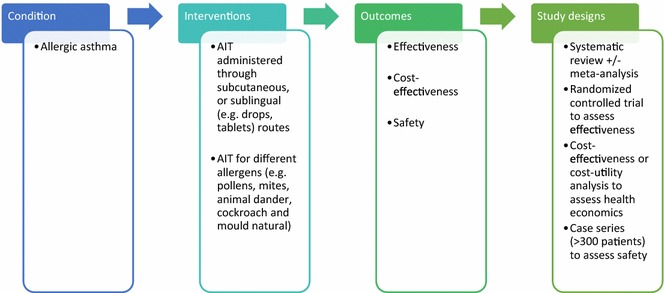
Conceptualization of systematic review of allergen immunotherapy for allergic asthma

A highly sensitive search strategy has been developed, and validated study design filters will be applied to retrieve articles pertaining to the use of AIT for allergic asthma from electronic bibliographic databases. We have conceptualized the search to incorporate the four elements shown in Fig. 1.

To retrieve systematic reviews, we will use the systematic review filter developed at McMaster University Health Information Research Unit (HIRU) (http://hiru.mcmaster.ca/hiru/HIRU_Hedges_MEDLINE_Strategies.aspx#Reviews). To retrieve randomized controlled trials (RCTs), we will apply the Cochrane highly sensitive search strategy for identifying RCTs in MEDLINE [8]. To retrieve case series, we will use the filter developed by librarians at Clinical Evidence: http://clinicalevidence.bmj.com/x/set/static/ebm/learn/665076.html.

We will search the following databases:

- Cochrane Library including,
  - Cochrane Database of Systematic Reviews (CDSR)
  - Database of Reviews of Effectiveness (DARE)
  - CENTRAL (Trials)
  - Methods Studies
  - Health Technology Assessments (HTA)
  - Economic Evaluations Database (EED)
- MEDLINE (OVID)
- Embase (OVID)
- CINAHL (Ebscohost)
- ISI Web of Science (Thomson Web of Knowledge)
- TRIP Database (www.tripdatabase.com)
- Clinicaltrials.gov (NIH web).
- Current controlled trials (www.controlled-trials.com)
- Australian and New Zealand Clinical Trials Registry (http://www.anzctr.org.au)

The search strategy has been developed on OVID MEDLINE and then adapted for the other databases (see Additional file 1: Appendix S1). In all cases, the databases will be searched from inception to October 31, 2015. Additional references will be located through searching the references cited by the identified studies, and unpublished work and research in progress will be identified through discussion with experts in the field. We will invite experts who are active in the field from a range of disciplines and regions to add to the list of included studies by identifying additional published and unpublished papers they are aware of and research in progress. There will be no language restrictions employed; where possible, all relevant literature will be translated into English.

### Inclusion criteria

#### Patient characteristics

We will focus on studies conducted on patients of any age with a physician confirmed diagnosis of allergic asthma, plus evidence of clinically relevant allergic sensitization as assessed by an objective biomarker (e.g., skin prick test or specific-IgE), in combination with a history of asthma symptoms due to allergen exposure.

#### Interventions of interest

This review is focused on AIT for different allergens (e.g. pollens, house dust mites, animal dander, cockroach and moulds), administered through either subcutaneous (SCIT) or sublingual (SLIT) routes compared with placebo or any active comparator.

#### Study designs

Systematic reviews of RCTs and RCTs will be used to investigate effectiveness; health economic analysis will be used to assess cost-effectiveness; and systematic reviews, RCTs and case series, with a minimum of 300 patients, will be used to assess safety.

#### Study outcomes

Primary

- Effectiveness (both short-term and long-term, where long-term is defined as persistence of benefit after discontinuation of AIT) assessed by symptom and medication scores.

Secondary

- Asthma control
- Asthma specific quality of life
- Exacerbations
- Lung function
- Environmental exposure chamber or bronchial allergen challenge
- Safety as assessed by local and systemic reactions
- Health economic analysis from the perspective of the health system/payer.

### Exclusion criteria

The following exclusion criteria will be applied:

- Reviews, discussion papers, non-research letters and editorials
- Animal studies
- Quantitative studies not employing systematic review or RCT techniques
- Qualitative studies
- Case series (less than 300 patients).

### Study selection

All references will be uploaded into the systematic review software Distiller and undergo initial deduplication. Study titles will be independently checked by two reviewers according to the above selection criteria and categorized as: included, not included or unsure. For those papers in the unsure category, we will retrieve the abstract and re-categorize as above. Any discrepancies will be resolved through discussion and, if necessary, a third reviewer will be consulted. Full text copies of potentially relevant studies will be obtained and their eligibility for inclusion independently assessed. Studies that do not fulfil all of the inclusion criteria will be excluded.

### Quality assessment strategy

Quality assessments will independently be carried out on each study by two reviewers using the relevant version of the Critical Appraisal Skills Programme (CASP) quality assessment tool for systematic reviews and economic evaluations [9, 10]. RCTs will be assessed for generation of allocation sequence, concealment of allocation, baseline outcome measurements, baseline characteristics, incomplete outcome data, blinding of outcome assessor, protection against contamination, selective outcome reporting and other risks of bias using the Cochrane risk of bias tool. Similarly, we will use the quality assessment form produced by the National Institute for Health and Clinical Excellence (NICE) to critically appraise case series [11]. Any discrepancies will be resolved by discussion or, if agreement cannot be reached, a third reviewer will arbitrate.

### Data extraction, analysis and synthesis

Data will be independently extracted onto a customized data extraction sheet in Distiller by two reviewers, and any discrepancies will be resolved by discussion or, if agreement cannot be reached, by arbitration by a third reviewer.

A descriptive summary with summary data tables will be produced to summarize the literature. If clinically and statistically appropriate, meta-analysis using either fixed-effect or random-effects modeling will be undertaken [8]. A narrative synthesis of the data will also be undertaken.

### Sensitivity and subgroup analyses, and assessment for publication bias

Sensitivity analyses will be undertaken by comparing the summary estimates obtained by excluding studies judged to be at high risk of bias with those judged to be at low or moderate risk of bias.

Subgroup analyses will be undertaken to compare:

- Children (5–11 years) versus adolescents (12–17 years) versus adults (≥18 years)
- SCIT versus SLIT AIT
- Monosensitized and mono-allergic versus polysensitized
- Mild/moderate versus severe disease.
- Treatment duration: ≤3 versus >3 years.

Where possible, publication bias will be assessed through the creation of funnel plots, and tested by Egger’s regression test and Begg’s rank correlation test [12, 13].

### Registration and reporting

This review will be registered with the International Prospective Register of Systematic Reviews (PROSPERO): http://www.crd.york.ac.uk/prospero/. The Preferred Reporting Items for Systematic Reviews and Meta-Analyses (PRISMA) checklist will be used to guide the reporting of the systematic review: http://www.prisma-statement.org/.

## Discussion

This review will involve systematically identifying, critiquing and synthesizing the evidence on the effectiveness, cost-effectiveness and safety of AIT for the management of allergic asthma. The findings from this review will be used to inform the development of recommendations for EAACI’s Guidelines on AIT. We anticipate that this review will report in 2016.

## Additional file

**Additional file 1: Appendix S1.** Search strategy.

## References

[1] The global asthma report 2014. http://www.globalasthmareport.org/burden/burden.php.

[2] World Health Organization (2007). Global surveillance, prevention and control of chronic respiratory diseases: a comprehensive approach.

[3] Papadopoulos NG, Arakawa H, Carlsen K-H, Custovic A, Gern J, Lemanske R (2012). International consensus on (ICON) pediatric asthma. Allergy.

[4] Haldar P, Pavord ID, Shaw DE, Berry MA, Thomas M, Brightling CE (2008). Cluster analysis and clinical asthma phenotypes. Am J Respir Crit Care Med.

[5] Hinks T, Zhou X, Staples K, Dimitrov B, Manta A, Petrossian T (2015). Multidimensional endotypes of asthma: topological data analysis of cross-sectional clinical, pathological, and immunological data. Lancet.

[6] SIGN BTS asthma guidelines 2012. https://www.brit-thoracic.org.uk/document-library/clinical-information/asthma/btssign-asthma-guideline-2014/. Accessed on 23 Sept 2015.

[7] Abramson MJ, Puy RM (2003). Weiner JM Allergen immunotherapy for asthma. Cochrane Database Syst Rev.

[8] Higgins JPT, Green S (editors). Cochrane handbook for systematic reviews of interventions version 5.1.0 [updated March 2011]. The Cochrane Collaboration, 2011. www.cochrane-handbook.org. Accessed on 3 Sept 2015.

[9] CASP checklist for systematic reviews. http://media.wix.com/ugd/dded87_a02ff2e3445f4952992d5a96ca562576.pdf. Accessed on 13 Nov 2015.

[10] CASP checklist for economic evaluations. http://media.wix.com/ugd/dded87_3b2bd5743feb4b1aaac6ebdd68771d3f.pdf. Accessed on 3 Sept 2015.

[11] Higgins JPT, Green S. Cochrane handbook for systematic reviews of interventions. Version 5.0.2 (Chapter 11, Section 11).

[12] Egger M, Davey Smith G, Schneider M, Minder C (1997). Bias in meta-analysis detected by a simple, graphical test. BMJ.

[13] Begg CB, Mazumdar M (1994). Operating characteristics of a rank correlation test for publication bias. Biometrics.

