# Erratum to: Allergen immunotherapy for allergic asthma: protocol for a systematic review

**DOI:** 10.1186/s13601-017-0167-6

**Published:** 2017-09-15

**Authors:** Sangeeta Dhami, Ulugbek Nurmatov, Ioana Agache, Susanne Lau, Antonella Muraro, Marek Jutel, Graham Roberts, Cezmi Akdis, Matteo Bonini, Moises Calderon, Thomas Casale, Ozlem Cavkaytar, Linda Cox, Pascal Demoly, Breda Flood, Eckard Hamelmann, Kenji Izuhara, Ömer Kalayci, Jörg Kleine-Tebbe, Antonio Nieto, Nikolaos Papadopoulos, Oliver Pfaar, Lanny Rosenwasser, Dermot Ryan, Carsten Schmidt-Weber, Stan Szefler, Ulrich Wahn, Roy-Gerth van Wijk, Jamie Wilkinson, Aziz Sheikh

**Affiliations:** 1 Evidence-Based Health Care Ltd, Edinburgh, UK; 2 Systematic Review at Decision Resources Group Abacus International, Oxford, UK; 3 Department of Allergy and Clinical Immunology, Faculty of Medicine, Transylvania University Brasov, Brasov, Romania; 4 Department of Pediatric Pneumology and Immunology, Charité Medical University, Berlin, Germany; 5 Food Allergy Referral Centre Veneto Region, University Hospital of Padua, Padua, Italy; 6 Wroclaw Medical University, Wrocław, Poland; 7 The David Hide Asthma and Allergy Research Centre, St Mary’s Hospital, Newport Isle of Wight, NIHR Respiratory Biomedical Research Unit, University Hospital Southampton NHS Foundation Trust, Southampton, UK; 8 Faculty of Medicine, University of Southampton, Southampton, UK; 9 Swiss Institute for Allergy and Asthma Research, Davos, Switzerland; 10 Sapienza University Rome, Rome, Italy; 11 National Heart and Lung Institute, Imperial College, London, London, UK; 12 University of South Florida, Tampa, FL USA; 13 Department of Allergy and Clinical Immunology, Sami Ulus Maternity and Children Training and Research Hospital, Ankara, Turkey; 14 Nova Southeastern University, Fort Lauderdale, FL USA; 15 University and Hospital of Montpellier and Inserm Paris Sorbonnes, Montpellier, France; 16 European Federation of Allergy and Airways Diseases Patients Association, Brussels, Belgium; 17 Children’s Center Bethel, EvKB, Bieledelf, Germany; 18 Allergy Center, Ruhr-University, Bochum, Germany; 19 Saga Medical School, Saga, Japan; 20 Hacettepe University, Ankara, Turkey; 21 Allergy and Asthma Center Westend (AAZW), Berlin, Germany; 22 Children’s Hospital La Fe, Valencia, Spain; 23 Allergy Department, 2nd Pediatric Clinic, University of Athens, Athens, Greece; 24 Department of Otorhinolaryngology, Head and Neck Surgery, University Hospital, Mannheim, Mannheim, Germany; 25 Center for Rhinology and Allergology, Wiesbaden, Germany; 26 Children’s Mercy Hospital, Kentucky, MO USA; 27 University of Edinburgh, Edinburgh, UK; 28 Technische Univ and Helmholtz Center Munich, Munich, Germany; 29 Children’s Hospital Colorado, University of Colorado School of Medicine, Aurora, CO USA; 30 Department of Pediatric Pulmonology, Charite, Berlin, Germany; 31 Section of Allergology, Department of Internal Medicine, Erasmus MC, Rotterdam, The Netherlands; 32 Pharmaceutical Group of the European Union, Brussels, Belgium; 33 Allergy and Respiratory Research Group, The University of Edinburgh, Edinburgh, UK

## Erratum to: Clin Transl Allergy (2016) 6:5 DOI 10.1186/s13601-016-0094-y

Unfortunately this article [1] was published with an error in the Funding section. The BM4SIT project is not acknowledged. This section should be corrected to the below:

**Funding**

EAACI and the BM4SIT project (Grant Number 601763) in the European Union’s Seventh Framework Programme FP7.
