# Supplementary material for: Allergen immunotherapy for allergic asthma: protocol for a systematic review
Source: Clin Transl Allergy. 2016 Feb 9;6:5. doi: 10.1186/s13601-016-0094-y (PMC4746770; doi:10.1186/s13601-016-0094-y)
Supplement: Supplementary file 1 — Additional file 1: Appendix S1. Search strategy. [file 13601_2016_94_MOESM1_ESM.docx]

**Appendix S1:**

**Search strategy 1**

**(MEDLINE, EMBASE)**

1. **exp asthma/**
2. **asthma.mp.**
3. **asthmatic children.mp.**
4. **acute asthmatic attack.mp.**
5. **asthma control.mp.**
6. **asthma exacerbations.mp.**
7. **wheez*.mp.**
8. **respiratory hypersensitivity/**
9. **bronchial disorder.mp.**
10. **hyper-responsiveness wheez*.mp.**
11. **lung function.mp.**
12. **ventilatory function.mp.**
13. **FEV.mp.**
14. **FEF.mp.**
15. **FVC.mp.**
16. **PEF.mp.**
17. **bronchial hyperreactivity.mp.**
18. **airway hyperreactivity.mp.**
19. **bronchial responsiveness.mp.**
20. **airway responsiveness.mp.**
21. **or/1-20**
22. **exp Desensitization, Immunologic/**
23. **exp Immunotherapy/**
24. **desensiti?ation.mp.**
25. **(immunotherapy or allergen immunotherapy or oral immunotherapy).mp.**
26. **subcutaneous immunotherapy.mp.**
27. **sublingual immunotherapy.mp.**
28. **specific immunotherapy.mp.**
29. **hyposensiti?ation**
30. **Or/22-29**
31. **exp Intervention Studies/**
32. **intervention studies.mp.**
33. **exp Clinical Trial/**
34. **(trial or clinical trial).mp.**
35. **Exp Randomized Controlled Trial/**
36. **randomi?ed controlled trial.mp.**
37. **exp Placebos/**
38. **placebos.mp.**
39. **exp Random allocation/**
40. **random allocation.mp.**
41. **random*.mp.**
42. **exp Double-blind method/**
43. **double-blind method.mp.**
44. **double-blind design.mp.**
45. **exp Single-blind method/**
46. **single-blind method.mp.**
47. **single-blind design.mp.**
48. **triple-blind method.mp.**
49. **search:.tw.**
50. **review.pt.**
51. **systematic review.tw.**
52. **meta analysis.mp,pt.**
53. **case series.mp.**
54. **(case$ and series).tw.**
55. **cost:.mp.**
56. **cost effective:.mp.**
57. **cost utility:.mp.**
58. **exp Health Care Costs/**
59. **(costs and costs analysis).mp.**
60. **economic evaluation*.mp.**
61. **((cost effective* adj1 analys*) or cost minimi?ation analys* or cost benefit analys* or cost utility analys* or cost consequence analys* or finances).mp.**
62. **Or/31-61**
63. **21 and 30 and 62**

**Search strategy 2**

**(Cochrane library, HTA, EED, CINAHL, ISI Web of Science, TRIP)**

**(Asthma or acute asthmatic attack or wheez* or respiratory hypersensitivity or bronchial disorder or hyper-responsiveness wheez* or lung function or ventilatory function or bronchial hyperreactivity or airway hyperreactivity or bronchial responsiveness or airway responsiveness)**

**AND**

**(Immunologic, desensiti* or immunotherapy or oral immunotherapy or allergen immunotherapy or specific immunotherapy or subcutaneous immunotherapy or sublingual immunotherapy or hyposensiti*)**

**AND**

**(Intervention stud* or experimental stud* or trial or clinical trial* or randomi* controlled trial or random allocation or single blind method or double blind method or triple blind method or random* or systematic review or meta-analysis or case series or economic evaluation* or cost effective* analys* or cost minimi?ation analys* or cost benefit analys* or cost utility analys* or cost consequence analys* or finances)**
